# Therapeutic efficacy of cancer stem cell vaccine

**DOI:** 10.1186/2051-1426-3-S2-P29

**Published:** 2015-11-04

**Authors:** Qiao Li, Yangyang Hu

**Affiliations:** 1 University of Michigan Medical Center, Ann Arbor, MI, USA; 2 University of Michigan, Ann Arbor, MI, USA

## Background

The inability to target cancer stem cells (CSC) may be a significant factor contributing to treatment failure. Novel therapeutic strategies are needed to immunologically target CSCs.

## Methods

We have developed a strategy to target the CSC populations in melanoma and squamous cell carcinoma using CSC lysate-pulsed dendritic cells (DC). D5 is a poorly immunogenic clone of the melanoma cell line B16 syngeneic to B6 mice. In minimal tumor model, B6 mice were inoculated subcutaneously with D5 cells. The 1st vaccine was administered 24 hours after tumor inoculation for treatment, followed by a 2nd vaccine on day 8. At the end of the experiments, the lungs were harvested and fixed with 10% formalin, paraffin embedded and stained with H&E to observe the histo-pathological alterations under the microscope. Freshly harvested primary subcutaneous tumors were disaggregated into single cell suspensions. Tumor cells were incubated with PE-anti-CCR7 and CCR10 or isotype controls. The cells were then resuspended in 2% formalin for flow cytometry analysis. The mRNA levels of chemokine CCL21, CCL27 or CCL28 in lung tissues were analyzed using real time quantitative PCR (qRT-PCR). Sorted ALDH^low^ or ALDH^high^ D5 cells were incubated with the immune supernatants collected from the cultured B cells with equal quantity of IgG followed by incubation with the 2nd antibody FITC-conjugated anti-mouse IgG. The binding of supernatant antibody to ALDH^low^*vs*. ALDH^high^D5 cells was assessed using flow cytometry. Survival analysis was determined by the log-rank test. Analysis for the presence of lung metastasis was performed using the Fisher exact test. Other data were evaluated by unpaired Student's t-test (2 cohorts) or one-way analysis of variance (ANOVA) (> 2 cohorts). A two-tailed P value

## Results

Using mouse models we demonstrate that DC pulsed with CSCs enriched by virtue of their expression of the CSC marker ALDH (CSC-DC) significantly inhibited local tumor growth, reduced development of pulmonary metastases and prolonged survival in the minimal tumor models. The effect was associated with down regulation of CCR7 and CCR10 in tumor cells and decreased CCL27 and CCL28 expression in lung tissue. CSC-DC vaccine significantly reduced ALDH^high^ CSCs. Direct targeting of CSCs was demonstrated by specific binding of IgG produced by ALDH^high^ CSC-DC vaccine-primed B cells to ALDH^high^ CSCs, resulting in lysis of these target cells in the presence of complement. When administered in the adjuvant setting after surgical excision of the SCC7 bulk tumor mass, administration of SCC7 CSC-DC vaccine reduced development of local tumor recurrence as well as systemic disease and prolonged survival demonstrating the efficacy of this approach.

## Conclusions

These data suggest that the CSC-DC vaccine approach may be useful in the adjuvant setting where local and systemic relapse are high after surgery.

